# Role of platelets in neuroinflammation: a wide-angle perspective

**DOI:** 10.1186/1742-2094-7-10

**Published:** 2010-02-03

**Authors:** Lawrence L Horstman, Wenche Jy, Yeon S Ahn, Robert Zivadinov, Amir H Maghzi, Masoud Etemadifar, J Steven Alexander, Alireza Minagar

**Affiliations:** 1 Wallace Coulter Platelet Laboratory, Division of Hematology and Oncology, Department of Medicine, Miller School of Medicine, University of Miami, Miami, Florida, USA; 2 Buffalo Neuroimaging Analysis Center, The Jacobs Neurological Institute, Department of Neurology, School of Medicine and Biomedical Sciences, State University of New York at Buffalo, Buffalo NY, USA; 3 Department of Neurology, Isfahan University of Medical Sciences, Isfahan, Iran; 4 Department of Cellular and Molecular Physiology, Louisiana State University Health Sciences Center, Shreveport, LA 71130, USA; 5 Department of Neurology, Louisiana State University Health Sciences Center, Shreveport, LA 71130, USA

## Abstract

**Objectives:**

This review summarizes recent developments in platelet biology relevant to neuroinflammatory disorders. Multiple sclerosis (MS) is taken as the "Poster Child" of these disorders but the implications are wide. The role of platelets in inflammation is well appreciated in the cardiovascular and cancer research communities but appears to be relatively neglected in neurological research.

**Organization:**

After a brief introduction to platelets, topics covered include the matrix metalloproteinases, platelet chemokines, cytokines and growth factors, the recent finding of platelet PPAR receptors and Toll-like receptors, complement, bioactive lipids, and other agents/functions likely to be relevant in neuroinflammatory diseases. Each section cites literature linking the topic to areas of active research in MS or other disorders, including especially Alzheimer's disease.

**Conclusion:**

The final section summarizes evidence of platelet involvement in MS. The general conclusion is that platelets may be key players in MS and related disorders, and warrant more attention in neurological research.

## Introduction

Platelets have long been implicated, or at least been suspected, in the etiology of a variety of neuropathologies, most obviously including ischemic stroke but also others such as multiple sclerosis (MS). In recent decades, a series of discoveries have been made which place those conjectures on a sound rational footing. Broadly speaking, the essence of these findings is that platelets possess an unexpectedly large variety of receptors and secretory products, additional to those serving their classical role in hemostasis and thrombosis, which are active in inflammation, immunity, and tissue repair. This versatility is remarkable in view of their very small size and lack of cell nuclei. Indeed, in the early days they were regarded as nothing more than cellular debris. These recent advances, together with the fact that platelets are often the first cells to arrive at sites of vascular injury, suggest the hypothesis that they may be central players in neurodegenerative diseases.

As the title states, this review provides a wide-angle perspective on platelets as mediators of inflammation and immunity, with emphasis on neurological implications. Therefore, it is not possible to treat each topic in the depth it deserves. Most of the topics are large and specialized fields in themselves with their own wealth of literature. However, the references supplied will lead the interested reader to more comprehensive accounts. Some good reviews of platelets in inflammation are available [1] but the present review is more wide-ranging and exhibits the relevance to neurology specifically at every opportunity.

## Platelet basics

Platelets, properly termed *thrombocytes*, were traditionally considered to function exclusively in hemostasis and thrombosis, a role for which they are superbly adapted. Platelets are produced as fragments of megakaryocytes and, according to the convincing arguments of Martin [2], this fragmentation occurs during passage through the lungs. Like erythrocytes, they lack nuclei but unlike erythrocytes they do possess mitochondria. They are about 1/4 the diameter of erythrocytes and about 1/24 as numerous, but they preferentially circulate along the vessel wall [3], positioning them to respond immediately to vascular injury.

All blood cells undergo activation in response to a rise in intracellular calcium but platelets are most spectacular in this response, transforming irreversibly within seconds from the resting discoid shape to extend numerous pseudopodia, and becoming highly adhesive to each other (aggregation), to other cells, and to almost any surface, notably on collagen exposed in the sub-endothelium, to form a platelet plug (white clot) [4]. However, weak agonists can induce partial and reversible activation ("priming"), which can be augmented synergistically by other agents *via *multiple pathways, and this primed state has been considered a target for drug intervention [5]. Platelet activation is also accompanied by secretion, i.e. the release of numerous substances from specialized granules, as listed in Table 1 and a list of acronyms and alternative names of various substances related to platelets is presented in Table 2. A list of major platelet glycoproteins is given in Table 3 and Table 4 lists the principal natural agonists of platelets and the receptors on which they act.

**Table 1:** Main Constituents of Platelet Secretory Granules

**Dense Granules**	
Solutes	5HT, ADP, ATP, GDP, GTP, Ca, Mg, PyroPi, histamine
Membrane	CD62P, CD63, GP's Ib & IIb/IIIa, LAMP2, Src, Ral-1
**Alpha Granules**	
Adhesive glycoproteins	fibronectin, vitronectin, vWF, TSP
Proteoglycans	PF4, ßTG, serglycin, HRGP & ßTG Ag's: PBT, CTAP-III, NAP-2
Mitogens:	PDGF, TGFß, ECGF, EGF, VEGF, VPE, IGF, IL-ß
Protease inhib's	TFPI, PAI-1, PDCI, α_2_-antiplasmin, C1 inhibitor, α_2_-antitrypsin, α_2_-macroglobulin
Coagulation:	Factors V, XI, XIII, HMWK, fibrinogen, PAI-1, protein C, protein S, protein C inhibitor, TSP-1, TSP-2
Membrane*	CD9, CD31, CD36, CD62P, CD144, GLUT-3,
* Not including several GP's found also on plasma membrane	
Misc.	IgG, IgM, IgA, albumin, GP Ia/multimerin, osteonectin, clusterin, angiostatin, endostatin, plasminogen
**Lysosomal Granules (Lysosomes)**	
Acid proteases	cathepsins D & E, carboxypeptidases A & B, collagenase, acid phosphatase, aryl sulfatase
Glycohydrolases	heparinase, b-galactosidase, b-glucuronidase, b-N-acetyl-glucosaminodase, b-glycerophosphatse, b-D-glucosidase, a-D-glucosidase, a-L-fucosidase, b-D-fucosidase, a-L-arabinosidase, a-D-mannosidase
Membrane	LIMP-1, LAMP-1, -2
**Dense Tubules**	Principal Ca store for internal secretion

Contents of the three main granules (dense, alpha, lysosomal) and the "dense tubules" are listed in Table 1.

**Table 2:** Main Constituents of Platelet Secretory Granules

Acronyms			
5HT	serotonin; 5-hydroxytryptamine	NAP-2	neutrophil activating peptide-2
ADP, ATP	adenosine diphosphate, -triphosphate	PADGEM	GMP-140. Former names for CD62P
CD144	Glut 3	PAI-1	plasminogen activator inhibitor 1
CD36	GP IV	PBP	platelet basic protein
CD62P	P-selectin; see also PADGEM	PDCI	platelet-derived collegenase inhibitor
CTAP-III	connective tissue activating protein III	PDGF	platelet-derived growth factor
EC	endothelial cell	PDGF	platelet-derived growth factor
ECGF	endothelial cell growth factor	PECAM	CD31; platelet-EC adhesion molecule
EGF	epidermic growth factor	PF4	platelet factor 4
Gbp	guanine binding protein	ßTG	beta thromboglobulin
GDP, GTP	guanine diphosphate, -triphosphate	TFPI	tissue factor pathway inhibitor
HMWK	high molecular weight kininogen	TGFß	transforming growth factor ß
HRGP	histidine-rich glycoprotein	TSP	thrombospondin
IGF	insulin-like growth factor	VEGF	vascular endothelial growth factor
LAMP1, 2	lysosomal associated membrane prot	VPF	vascular permeability factor
LIMP, CD63	lysosomal integral membrane protein	vWF	von Willebrand factor
NAP-2	neutrophil activating peptide-2		

The standard abbreviations used are spelled out in Table 2. The contents of each are classified in accordance with most of the sources. The main source was Ch. 7 of Gresele et al [11], with some updates from other sources such as [1]. Similar tables appear are found in textbooks of hematology but details may differ. For example, Table twenty four to three of Colman et al [10] lists about twice as many acid hyrdrolases of the lysosomes. No attempt was made to give a complete listing, i.e. many agents cited in text are not listed here. More details can be found in more recent or specialized sources such as [318]. The subcellular localization of some recently identified secreted substances may not yet be known. Many proteins of the platelet plasma membrane are also found on the membranes of granules and in the invaginations of the membrane known as the *open canalicular system *(OCS).

**Table 3:** Platelet surface glycoproteins (GP's) and agonists

		Major Platelet Glycoprotein (GP) Receptors	
Classical	CD #	Integrin	Activating Ligand(s)
GP IIb/IIIa	CD41b	α_IIb_ß_3_.	Fibrinogen, vWF, fibronectin, vitronectin
GP Ib-IX	CD42a,b,c		vWF, thrombin
GP Ia-IIa	CD49b	α_2_ß_1_.	Collagen
GP Ic-IIa	CD49e	α_5_ß_1_.	Fibronectin
GP Ic-IIa	CD49f	α_6_ß_1_.	Laminin
Vitronectin		α_V_ß_3_.	Vitronectin, vWF, fibronectin, fibrinogen, TSP
PECAM-1	CD31		Further interaction with endothelium
P-selectin*	CD62P		Interaction with leukocytes
* a.k.a. PADGEM or GMP-140 in the older literature.			
Listed: Ib, IIa, IIb, IIIa, IIIb (a.k.a. GP IV, CD36), V, IX.			

The classical GP designations (which are based on location in electrophoresis) are given in the first column and the antigen CD numbers in the 2nd column. The 3rd column gives the newer integrin names, which are compounded of two subunit types (α, ß). Integrin nomenclature is awkward, hence the classical names remain in wide use. Many of the GP's are known also by other names, e.g. VLA-2, -3, -5, -6 (for "very late antigens") corresponding respectively to integrins α_2_ß_1_, α_3_ß_1_, α_5_ß_1 _(or α_V_ß_1_), and α_6_ß_1_. The 4th and last column gives the ligands which activate platelets *via *those GP receptors, and if more than 1 is given, they are in order of potency. For example, the complex GP Ib-IX is the main site for vWF but it has some activity for other GP sites, and likewise collagen. In most cases there is evidence of receptor mobility, clustering, interaction, synergy and inter-dependence, as noted for Table 4. Data adapted from Table 22.1 and others in Colman et al [10].

**Table 4:** Platelet surface glycoproteins (GP's) and agonists

	Main Platelet Agonists						
	Agonist	Receptor(s)		Integrin(s)		For GPCR's:	
Strongest	Thrombin + collagen						
	A23187*			-			
Strong	Thrombin	PAR1	PAR4	α_IIb_β_3_	α_2_β_1_		
	Collagen						
	PAF						
Medium	ADP	P2Y_1_	P2Y_12_			G_qα_	
	TXA_2_.	TP-α, -ß				G_q_	G_12_
Weak	Epinephrine	α_2A _AR					
	Arachidonate						
	Vasopressin						
	Serotonin						
Damping	Prostacyclins	IP R	EP_2 _R				
	PACAP, VIP	VPAC R				G_sα_	
* A23187, calcium ionophore [non-physiological]; admits external calcium.							

Listing of the main primary platelet agonists in approximate order of potency, and their receptors types, e.g. PAR = *P*rotease-*A*ctivated *R*eceptor. Other abbreviations are listed below. By "primary" is meant having direct action. For example, fibrinogen and vWF (Table 1) do not bind or activate the normal resting platelet. The third column lists the integrin names and the last column indicates some which are known to be GPCR's (= *G*-*P*rotein *C*oupled *R*eceptor). Similar listings appear in many sources. The relative potency may be misleading since it is based on data *in vitro *whereas *in vivo *several of these agonists, especially the "weak" ones, can act synergistically to potentiate or "prime" responses to others [5]. For example, serotonin [266,319]; and vWF-induced aggregation requires co-stimulation (including by ristocetin, an antibiotic used in laboratory studies) or conformation change of vWF. Information on the GPCR's is simplified from Ch. 107 of Hoffman et al [8] with terminology updated according to Van Geet et al [320]. The "damping" agonists shown at bottom inhibit activation by raising cAMP (cyclic AMP) levels, which is opposite the usual effect of cAMP elevation in other cells. These tables are for orientation only. *Other abbreviations (see also Table 2)*: TXA_2 _= thromboxane A_2_; α_2A _AR = a_2A_-adrenergic receptor; PACAP = pituitary adenylyl cyclase activating peptide; VIP = vasointestinal peptide; prostacyclins refer to prostaglandins such as PGE_1_, PGI_2_.

Equally important is the ability of activated platelets, and microparticles from them which are released concomitantly, to catalyze the coagulation cascade. This is accomplished by an activation-dependent membrane inversion or "flip-flop" by which normally in-facing phospholipids (PL) become exposed to plasma [6]. These PLs are mainly anionic, such as phosphatidylserine (PS), and render the membrane procoagulant by promoting assembly of the vitamin K-dependent coagulation factors into their active complexes (prothrombinase and factor X-ase), to generate the thrombin that converts fibrinogen to the fibrin plug. The role of vitamin K in this process is to serve as a cofactor for the post-translational addition of an extra carboxy group to glutamic acid residues (Gla domains), enabling them to bind calcium, much as citrate does, thereby anchoring the coagulation factors to suitable PL membrane surfaces (such as PS) *via *calcium bridging.

Since hemostasis and thrombosis is not the focus of this article, readers wishing more depth are referred to standard textbooks of hematology [7-10] or more specialized books [11].

## Introduction to platelets as inflammatory mediators

It had become clear by 1995 that platelets also play major roles in inflammation and immunity [12-15]. This concept was amply confirmed and extended by subsequent findings [16], beginning with the discovery around the same time of the first chemokine receptor on platelets (referenced below). The following paragraphs briefly review the major classes of platelet-derived factors which are active in inflammatory states, with emphasis on implications for neurological disorders.

## Matrix metalloproteinases in platelets

The matrix metalloproteinases (MMPs) are a group of about 40 homologous proteases which are secreted to the extra-cellular matrix (ECM) in an inactive form. As a group these enzymes depend on a metal ion for activity, generally zinc. A subgroup of these enzymes, the membrane-type (MT-MMP), remains at the plasma membrane and can activate the secreted forms of the enzyme. Other closely related enzymes are those of the ADAM's (*A D*istintegrin *A*nd *M*etalloprotease) and the ADAMTS's (ADAM with *T*hrombo*S*pondin domain) [17,18]. Many are also known by their earlier common names. They have varying degrees of substrate specificity towards many proteins in the vicinity of the ECM including collagen, fibronectin, gelatin, laminin and other stromal components ("stromelysins"). Accordingly, their activities must be finely controlled and regulated by the aforementioned activators, specific inhibitors known as TIMP's (Tissue Inhibitor of *MMP*), and by the circulating plasma protein, alpha-2-macroglobulin. MMPs are important in many aspects of human development and tissue remodeling, and play significant roles in pathogenesis of neuroinflammatory disorders.

MMPs have been generally recognized as major participants in disruption of the blood-brain barrier (BBB) in MS [19] and there is persuasive evidence that they play a direct causal role [20]. This evidence consists of close association of rising levels, particularly of MMP-9, prior to onset of exacerbations in humans and in the animal model of MS known as *experimental autoimmune encephalomyelitis *(EAE) [21-23]. Relative reduction of TIMP's was also seen. In EAE model, disease severity is sharply attenuated by inhibition of MMP or by gene knockout. Similar results were observed with an alternative model of MS, demyelinating canine distemper virus infection [24]. In human immunodeficiency virus (HIV) infection, MMP-9 in particular was implicated in disruption of the BBB [25]. MMPs have also been implicated in post-ischemic brain injury [26]. MMP-9 may be particularly noxious [27] but MMP-2 is also discussed as a biomarker [28], as are others for the case of MS [20]. The mechanism usually proposed is that infiltrating leukocytes employ MMPs to disintegrate the basement membranes of cerebral endothelial cells to enter the CNS.

Accordingly, inhibitors of MMPs are under active investigation for treatment of MS and some other neuropathologies [29]. At least one action of tetracycline derivatives such as minocycline and doxycycline is inhibition of MMPs [30-32]. Indeed, interferon-beta1a (IFN-beta) is reported to have such action [33,34]. On the other hand, several groups caution against prolonged and non-specific inhibition of MMPs for therapy because of their equally prominent role in repair, recovery and normal CNS health [35-37].

How might platelets be placed in these events? First, platelets have been shown to express MMPs -1, -2, -3, -9; ADAM-10, -17; all four TIMP's except TIMP-3; MT1-MMP (also known as MMP-14); and ADAMTS-13 [38,39]. The cited review [38] is up-to-date but limits discussion to the role of MMPs in platelet function and does not explore the likely effects of MMPs secreted during platelet activation on bystander cells, analogous to the manner in which neutrophils or glial cells can injure neurons in the vicinity [40]. Second, platelets are often the first responders to sites of injury or inflammation. Third, it is known that platelets can interact with leukocytes to form circulating complexes, first demonstrated by Rinder et al [41,42], extended by our laboratory to platelet microparticles (PMP) [43], and now widely employed as an assay of activated or inflammatory states, e.g. [44-47]. It has been shown that formation of platelet-leukocyte complexes is associated with the expression and activation of MMPs -1, -2, -3 and -9 [48]. It is not clear in that paper if the source of MMP is platelets or leukocytes (it is likely both) but inhibitors of MMP in the media reduced formation of the complexes while active MMP promoted them.

Fourth, is the effect of platelet-derived microparticles (PMP). Although little work of a specifically neurological nature has been done in this area, cancer researchers have demonstrated that PMP strongly promote the invasive potential of prostate and breast cancer cells in a manner dependent on MMP expression [49-51], a process resembling leukocyte infiltration to the CNS. PMP express most of the platelet membrane proteins, therefore likely including MT-1 MMP, which was notable in the study [51]. The work by Jy et al of our laboratory demonstrated that PMP can directly activate and bind to neutrophils [43]; however, MMP activities were not measured. We have recently presented evidence that cell-derived microparticles, especially PMP, may play an important role in neurodegenerative diseases [52,53]. Although studies of platelet activation in MS are rare in the recent literature, the data of Cananzi et al from patients in remission clearly demonstrate chronic platelet activation in MS [54] (their figure two), confirming earlier reports, which they cite, and which we discuss in the closing section of this paper.

## Platelet chemokines

Chemokines are central to inflammation chiefly by signaling leukocyte migration/infiltration and differentiation, but also by other actions [55]. About 50 are known and half as many receptors. They are low molecular weight proteins (7-12 kDa) and are known both by acronyms for their common names and by systematic nomenclature, the latter consisting of two main groups, CC and CXC, and a few exceptions. These symbols refer to the amino acids (aa), Cys-Cys or Cys-X-Cys where X is any other aa. Suffix L for "ligand" or R for "receptor" is applied. The alpha chemokines are the CXCL's and beta chemokines are the CCL's. In general, the CCL's bind only to CCR's, while CXCL's bind only to CXCR's. Beyond that, the receptors are promiscuous to varying degrees, suggesting complex and subtle signaling depending on relative concentrations and affinities of the ligands present.

The discovery of the first chemokine receptors on platelets around 1995 by Power et al [56-58] was a watershed in platelet biology. This was followed by a succession of advances (e.g. [59-61]), leading to the present knowledge of platelet chemokines, reviewed in 2003 [62], 2007 [63] and 2008 [64]. The 2007 review is particularly clear but each brings out novel points. Platelet factor 4 (PF4), now called CXCL4, was the first known chemokine (though not originally so named) and is secreted in abundance from the alpha granules, along with about 10 other platelet chemokines. In Table 5 a list of platelet chemokines and responses to them is presented. Most of the work on platelet chemokines has been orientated to cardiovascular disease. Useful insights might be gained from tissue co-culture of brain endothelia with astrocytes (model of BBB) to study the role of platelets in leukocyte adhesion, transmigration, and other phenomena relevant to neuroinflammatory diseases.

**Table 5:** Platelet chemokines and receptors

Platelet Chemokines and Receptors			
Platelet Chemokine	Common Name	Receptor(s)	Platelet Chemokine Receptors
CXCL1	GRO-α	CXCR2>CXCR1	CCR1
CXCL4	PF4	CXCR3B, GAG	CCR3
CXCL4L1	PF4alt	unknown	CCR4
CXCL5	ENA-78	CXCR2	CXCR4
CXCL7 [a]	NAP-2	CXCR2>CXCR1	CX3CR1
CXCL8	IL-8	CXCR1, CXCR2	
CXCL12	SDF-1α	CXCR4	
CCL2	MCP-1	CCR2	(Those which are disputed are not shown)
CCL3	MIP-1α	CCR1, 2, 3	
CCL5	RANTES	CCR1, 3, 4, 5	
CCL17	TARC	CCR4, CCR8	

Platelet chemokines and their common names are listed at left, then their receptors, not all of which are present on platelets. Those which are present on platelets are listed in the right-most column. As noted in text, there is considerable promiscuity of chemokines towards the receptors, some being more specific than others.

*Acronyms for common names of chemokines that activate platelets*: CCL17 (TARC): thymus and activating-regulating chemokine; CCL22 (MDC): macrophage-derived chemokine; CXCL12 (SDF-1): stromal cell-derived factor 1alpha; CCL18 (PARC): pulmonary and activation regulated chemokine.

Meanwhile, recent advances have created more complexity, not simplification. It should be noted that our knowledge of platelet chemokines and receptors for them is based largely on murine studies and tissue culture with limited cell types. For example, human umbilical vein endothelial cells (HUVEC) are widely used because they are easy to obtain and grow, but they often exhibit quite different responses compared to brain micovascular EC, as we have observed in microparticle studies [65] and pointed out for *in vitro *studies of antiphospholipid antibodies [66]. Endothelial cell (EC) activation is not a simple yes/no effect but a multi-pathway phenomenon having both immediate effects (within seconds of stimulation, such as CD62E cell surface expression) and slower effects (24-48 hr) that depend on genetic upregulation of the pathways activated by, for example, exposure to TNF-α [67,68].

Some unwarranted conclusions about platelet chemokines have been put forth. Geissner suggests [64] that only platelet CXCL4 (PF4) and CXCL7 are likely to be important since other cells are richer sources of the remaining platelet chemokines, and some platelet chemokines are acquired from plasma (Table 6). This implies that the other platelet chemokines are inconsequential, and overlooks the fact that platelets are often the first cells to arrive at a site of vascular injury, and are exceptionally responsive to stimuli. Therefore, platelets may indeed be an important source in the microenvironment of all chemokines they carry, as has been noted by Baltus et al for monocyte recruitment [69]. Full discussion of the platelet chemokine system is beyond the scope of this review, while our purpose is limited to raising awareness of the new face of platelet biology. However, to add dimension, notes on a few of the platelet chemokines follow. Chemokines involved with repair and recovery are considered in the next section. For surveys of chemokines particularly relevant to MS, see [70,71].

**Table 6:** Processing of CXCL7

	Processing of CXCL7
**pro-PBP**	pro-Platelet Basic Protein
↓	
**PBP**	Platelet Basic Protein
↓	
**CTAP-III**	Several actions
↓	
**β-TG**	Several actions
↓	
**NAP-2**	Most pronounced actions

This shows steps in the processing of CXCL7, which is unusual in having distinctive activities at each step.

### Rantes

This has long been recognized in platelets [60,72]. It was suspected of being carried on platelet microparticles [73] and that was subsequently confirmed [74]. Gene polymorphisms for this chemokine (CCL5) and one of its receptors (CCR5) affect the susceptibility, severity, and age of onset of MS [75]. Ubogu et al found that mononuclear cell migration across an *in vitro *model of the BBB was driven by CCL5, being inhibited by antibodies against it [76,77]. That leukocyte infiltration is driven by CCL5 gained further support by the clinical and laboratory observations of Jalosinski et al [78]. At least one novel drug is in the pipeline which targets CCR1 (another receptor for CCL5) for therapy of MS and other inflammatory disorders [79]. Similar findings on the importance of RANTES are seen in EAE models [80-83]. This sampler of literature makes clear that CCL5 is important in MS. However, the participation of platelets as source of these cytokines is not considered in these papers, nor are platelets present in the *in vitro *studies, though it is clear that platelets are potentially important players.

### Trem

This does not have the structure of a true chemokine receptor but is included here because of its role in inflammation. Named as the *T*riggering *R*eceptor *E*xpressed on *M*yeloid cells (TREM), it was discovered in 2000, was identified on platelets soon after, and is only recently being understood [84,85]. Initial reports were that it was pro-inflammatory, then TREM-2 was identified and appeared to act oppositely (anti-inflammatory). Current thinking is that all three known TREM's act to integrate many kinds of signals, in concert with DAP-12 as a complex. A soluble form also exists. The main known triggering ligand is the *T*REM-*L*ike *T*ranscript *1 *(TLT-1), which inhibits thrombin-induced platelet activation [86], is secreted from platelet α-granules [87], and modulates neutrophil activation [88] and probably other leukocytes. Because of its seeming role as a kind of "master integrator" of pro- and anti-inflammatory signals ("mixed messages"), it is of great interest in neuroinflammatory conditions such as MS, as referenced [85]. Here again, the possible contribution of platelets to TREM-mediated events is rarely mentioned.

### Platelet factor 4 (PF4; CXCL4)

PF4 was discovered early (1977) and is secreted in abundance from platelets upon activation. Accordingly, its measurement has been taken as an index of platelet activation, such as in MS [54]. As a chemokine, it is unusual in several ways. It does not exert chemotaxis for any cell yet tested but a large number of other activities have been attributed to it (see Table 2 of [63]). Indeed, the list of actions is so lengthy that some have doubted the specificity of PF4 effects. A possible solution to this "embarrassment of riches" is the hypothesis set forth by Sachais et al [89], that the true function of PF4 is not specific signaling but is to neutralize electric charge on glycosaminoglycans (GAG's), as this could well explain the majority of its reported actions. (Recombinant PF4 reached clinical trials as an alternative to protamine sulfate for neutralizing heparin [90].) Clinically, PF4 is best known as the target antigen of heparin-induced thrombocytopenia (HIT); that is, the IgG of HIT is directed against PF4, not heparin *per se *[91]. Intriguing parallels have been drawn between HIT and anti-phospholipid syndrome (APS) [92], and between APS and MS, as we have referenced [66].

## Platelet cytokines and growth factors

The cytokines constitute a lengthier list of signaling molecules, notably including the interleukins, and overlaps somewhat with the chemokines. Some helpful summary tables (such as distributed by R&D Systems) include both groups, and a number of members are commonly listed in both families, e.g. interleukin 8 (IL-8) is now CXCL8, and RANTES, once considered a cytokine, is now assigned as CCL5. Originally, a sharp distinction was drawn between factors that attracted leukocytes (chemokines) and factors with other effects (cytokines, e.g. the interleukins) but that distinction is increasingly blurry in view of the pleiotropic actions of so many of these substances.

As the first cells to arrive on the scene of vascular injury, it makes sense that platelets would be involved with tissue repair as well as plugging leaks. Accordingly, some of the most informative reviews of platelet cytokines are oriented to the role of platelets in wound healing [93-95]. One may suspect important roles in neurological tissue repair as well. The 2008 review by Nurden's group [94] includes dozens of factors in addition to the chemokines listed above. Although no attempt will be made to list them here, the platelet "growth factors" include EGF, TGF-β1, -β2, PDGF, HGF, FGFb (FGF-2), and a series of pro- and anti-angiogenic factors, e.g., VEGF-A, -C. Platelets also secrete the antimicrobial peptides known as *thrombocidins*. Of special interest in neurology is the presence of semaphorin 3A (a.k.a. collapsin-1), another of many "CNS-specific" agents found in platelets. Also listed in [94] are the cytokines TRAIL, LIGHT, SDF-1α, HMGB-1, etc., and the interleukins 1L-8 and IL-6sR in platelets. Their review misses a few, notably IL-1 from platelets [96,97], which is secreted on platelet microparticles. Further discussion of these platelet-derived agents (which are difficult to neatly classify) is beyond the scope of this review, our purpose being to bring to wider attention the astonishing variety of bioactive platelet-derived agents. The following few examples may not be widely appreciated.

### CD40 and CD40 ligand (CD40L, a.k.a. CD154)

Platelets are the main source of CD40L, secreting it to deliver co-stimulatory signals to antigen-presenting cells (APC's) [98-100]. Of note, platelets can induce maturation and activation of dendritic cells (DC) [101,102], but probably involving more than CD40L alone [103]. The role of CD40L in MS is well appreciated in reviews [104,105] and is a target of new therapies [106,107]. Filion et al report that CD40L levels on monocytes were highest in secondary progressive MS (SPMS) [108]. Harp et al demonstrated that CD40L/IL-4 as well as another stimulating reagent induced B cells to upregulate CD80 and HLA-DR; however, only CD40L/IL-4 was effective in eliciting CNS-antigen specific proliferation by autologous T cells [109]. CD40L is perhaps best known for other putative actions which make it a risk factor in cardiovascular disease [110], although somewhat controversially.

An important technical issue arises with CD40L. Prior to about 2005, and often yet today, circulating levels of CD40L were measured in serum by standard ELISA kits. We reported in 2004 [111] that serum levels are largely an artifact of platelet activation during blood clotting, being from 10-fold to 50-fold higher than in plasma specimens. This was subsequently confirmed by at least two other groups, and the kit makers have since changed their instructions to advise use of plasma, not serum for assay. In view of this, one must question the significance of reports based on serum assays, since serum levels reflect the total releasable platelet CD40L, not the true plasma level. It appears that this is not yet fully appreciated since several reviews make no mention of it and accept earlier reports on the same footing as more recent studies that measure plasma levels. Moreover, one may expect similar artifacts for other chemokines secreted from platelets if measured in serum, especially PF4, but also the others since nearly all will be released to serum as an artifact of clotting, inflating true plasma levels.

## Toll-like receptors on platelets

The discovery of toll-like receptors (TLRs) on platelets in 2004 was another completely unexpected development. Other immune functions of platelets had been earlier noted, including generation of killer-like reactive oxygen species (ROS) [13], phagocytic activity, secretable antimicrobials (thrombocidins), interactions with leukocytes and endothelial cells by direct contact or secretory signaling, and as the principal source of CD40L (CD154) [100,110].

First discovered in the fruit fly, about a dozen TLR's are now known and each is tuned to recognize a distinct class of pathogen-associated molecular patterns (PAMP's). The PAMP's which are recognized include bacterial components such as flagellin, lipopolysaccharide (LPS), lipoproteins, peptidoglycans, certain regions of DNA, and so on. The true TLR's are transmembrane surface proteins, but proteins with similar PAMP-recognition functions for virions occur in the cytoplasm [112,113]. The membrane TLR's, some of which function as heterodimers (e.g. TLR2 plus TLR1 or TLR6), upon engagement with ligand, pass their signals through a series of accessory transducers to the cell nucleus where antimicrobial genes are activated to yield products such as IL-1 and TNF-α. Since platelets lack nuclei, however, they seemed irrelevant to studies of the tissue distribution of TLR's.

To our knowledge, it was Shiraki et al who discovered the first TLR's in platelets, TLR1 and TLR6, by mRNA, Western blotting, and flow cytometry [114]. Furthermore, their expression was upregulated in response to IFN-γ. The next year, Cognasse et al reported also TLR2, TLR4, and TLR9 on platelets [115], and observed that expression levels increased two-fold upon platelet activation. More recently, the latter authors have followed up with further insights on platelet TLR's, and propose a major and unique role of platelets in bridging innate to adaptive immunity on this basis [116,117].

These findings add a new dimension to studies of TLR's in neuroinflammatory conditions. For example, the study by Chearwae and Bright of TLR4 and TLR9 in a model of MS documented upregulation of these receptors in T cells after induction of EAE, and favorable reduction along with amelioration of symptoms with prostaglandin and curcumin treatment [118]. However, now that platelets are known to also possess these receptors, it appears that their participation in such processes deserves consideration in future experiments of that kind.

## Peroxisome proliferator-activated receptors

Peroxisome proliferator-activated receptors (PPAR's), of which three are known, are ligand-activated nuclear transcription factors of the hormone receptor superfamily. They are widely disseminated and appear to function chiefly in regulating metabolism, notably of fats, and this fact has elevated the fibrate drugs (agonists of PPAR) to a level of importance second only to the statins for prevention and treatment of coronary artery disease. However, like the statins, they seem to have "pleiotropic" effects, prominently including anti-inflammatory actions now under investigation for treatment of MS [119-121]. Indeed, the role of PPAR's in CNS disorders is of much current interest [122,123], with promise of applications in Parkinson's and Alzheimer's diseases among others [124,125]. Yang et al has reported on PPARα regulation of immunity and the EAE model of MS [126]. PPAR's appear to be an important target of the NSAID's [127]. Interestingly, it appears that lysophophatidic acid (LPA) is a natural activator of PPAR [128], and that agonists of endocannabinoid receptors also stimulate PPAR in a model of MS [129]. Importantly for MS, PPAR appears to control inflammation induced by CD4+ T cell infiltration, at least *in vitro *[130].

Since platelets lack nuclei they are not expected to have PPAR's, but platelets are full of surprises. Both PPARβ/δ and PPARγ were found expressed in platelets [131]. Furthermore, the same authors have demonstrated that PPARγ is released from activated platelets on microparticles (PMP), which is taken up by promonocytic cell line in tissue culture; and that agonists of PPARγ induced platelet release as judged by secretion of sCD40L and thromboxane A_2 _(TxA_2_) [131]. Although the full implications of these late findings remains to be seen, they are paralleled by other developments in PPAR research, such as the recognition that active PPAR is not necessarily confined to the surface of the nucleus. The therapeutic potential of agonists of PPAR, such as rosiglitazone (Avandia™) and pioglitazone (Actos™) for PPARγ, needs further study but it has been shown that they inhibit platelet release of CD40L [131].

## Platelet-derived bioactive lipids

Recognition that certain lipids perform critical signaling functions began in the 1970's with the observation that human semen caused contraction of uterine muscle strips, leading to identification of the *prostaglandins*, so called for that reason. Related families of active lipids, such as the leukotrienes and thromboxanes, subsequently came to light and are collectively known as the *eicosanoids*, for the 18-carbon arachidonic acid precursor in the cell membrane. From the outset, platelets were seen as a major source of these agents. Indeed, a common measure of platelet activation today is the circulating level of thromboxane B_2 _(TXB_2_), the stable breakdown product of thromboxane A_2 _(TXA_2_, half-life 30 sec's). That early work culminated in understanding the mechanism of aspirin and led to development of the other COX inhibitors and NSAID's, most of which inhibit platelet activation. Since that work is well-known, we shall limit discussion to brief review of some of the more interesting recent developments relevant to the link between platelets and neuro-inflammatory diseases. Several recent reviews are available on the general subject of bioactive lipids [132] and with emphasis on the neuronal nucleus [133].

### Lysophosphatidic acid (LPA) and sphingosisine-1-phosphate (S1P)

The emerging significance of LPA in medicine has been reviewed [134,135] and with focus on autoimmune [136] and neurological diseases [137]. Its sphingolipid homolog, S1P, will not be reviewed here because it is well-known to neurologists as the target of FTY720 (fingolimod), a pro-drug that antagonizes the S1P receptor(s) to limit leukocyte migration for treatment of MS [138,139].

LPA is formed mainly by the enzyme, autotaxin [a.k.a. lysophospholipase D (lysoPLD)], which is secreted by platelets and other cells and is inhibited by LPA [140,141]. Most authors accept platelets as the main source of plasma LPA, and this is supported by a study of the effect of aspirin in cerebral vascular disease: aspirin treatment reduced LPA levels, which rose again when aspirin was stopped, leading the authors to conclude that platelet activation is the major source of LPA [142]. LPA can also be formed by mild oxidation of low-density lipoprotein (LDL) [143]. Of the several known LPA receptors, LPA (5) is established for platelets, is highly selective, and may be centrally involved in platelet activation [144].

LPA alone appears to be a weak agonist of platelets but its potency is increased synergistically by the presence of other lysolipids [145]. Similarly, Eriksson et al found synergy of epinephrine with LPA in inducing adhesion of platelets to an albumin-coated surface [146]. The study of Kang et al [145] documents augmented production of CXCL16, a regulator of T cell migration, by macrophages due to presence of LPA (or S1P) following stimulation by LPS, of interest in MS because of parallels with S1P. Also of interest *vis a vis *MS is involvement of LPA in lymphocyte-endothelial interaction in high endothelial venules [147], and in endothelial barrier function [135]. It is reported that PPARγ is an important target of LPA [141], wherefore those authors are developing inhibitors of the LPA receptor. It has been convincingly shown that LPA is also involved in the regulation of blood pressure [148].

Lin et al showed that LPA stimulated expression of IL-8 and monocyte chemo-attractant protein-1 (MCP-1) in endothelial cells, in a manner that depended on IL-1 [149]. It is not widely appreciated that platelets are an important source of IL-1, particularly in a localized micro-environment [150]. Several studies have found potentially important links between platelet-derived LPA and cancer metastasis [151,152].

Interestingly, it has been found that human platelet responses to LPA depend strongly on individual donors [153], classified by those authors as "responders" and "non-responders", and leading them to propose a novel inhibitory pathway in the ≈20% of non-responsive subjects. Another group also reported variations in observed effects depending on donor [146]. In assembling this review we noted some apparent conflicts on the reported action of LPA on human platelets, which are probably resolved in part by donor differences. Indeed, it was reported that in mice, LPA markedly *inhibited *platelet activation, induced a *bleeding *diathesis, and *attenuated *thrombosis [140]. Such opposite effects may be explained by alternative receptors [154] but the details are obviously complicated and poorly understood. Much remains to be clarified about the role of LPA in hemostasis and thrombosis, and in its many other putative roles in health and disease.

### Endocannabinoids

The endocannabinoids, such as 2-arachidonyl glycerol (2-AG), are lipid mediators discovered in 1995 which have recently shown promise against neuroinflammatory disorders, as in the virus EAE model of MS of Loria et al [155]. Mechanisms of this benefit are said to include activation of PPAR. With specific regard to platelets, a key enzyme involved was purified from platelets and studied [156], more recently in further detail [157]. There is no doubt that 2-AG can be produced by platelets, and that 2-AG induces platelet activation [158] but it is not yet clear if platelets possess one of the two receptors for 2-AG, dubbed CB(1) and CB(2), since those authors found no evidence for this receptor on platelets whereas Schafer et al found that a specific antagonist of CB(1), rimonabant, blocked the effect of 2-AG [159].

### Lipoxins; resolvins, protectins

These lipid mediators of inflammation are recently identified. The extent to which platelets contribute them has not (to our knowledge) been much studied but the fact that they are sensitive to aspirin [160] suggests platelets as a significant source, i.e., platelets are major players in the "inflammatory hypothesis" of cardiovascular disease [161]. The *resolvins *are named for their role in *resolving inflammation*. Being derived from omega-3 fatty acids *via *the lipoxygenase (LOX) pathway, this may offer a rational basis for the cardiovascular benefits of polyunsaturated fatty acids (PUFA's) [160,162,163]. Resolvins have been shown to inhibit reperfusion brain injury, and the brain lipid messenger, 10,17S-docosatriene, potently inhibited leukocyte infiltration and other negative measures [164]. Resolvin E1, derived from omega-3 eicosa fatty acid, inhibited platelets in an agonist-specific manner, reduced leukocyte rolling in venules of mice, and modulated expression of several adhesins in monocytes and neutrophils, possibly accounting for some of the benefits of dietary PUFA's [165]. Further work may lead to important new insights on lipid mediators in neurodegenerative diseases and their possible relationship to platelet activation [166-169].

### Platelet activating factor (PAF)

PAF is named for its potent activation of platelets, with effects down to picomolar concentrations [170]. It was discovered in the late 1970's by its strong anaphylactoid action, distinct from that of complement C3a, C5a [171], and was found to be a potent chemotactic stimulus for inflammatory cells [172,173]. Its biochemistry was well described by Braquet [174], who also described some synthetic inhibitors of it intended as drug candidates, and the natural inhibitors from the plant, *Gingko biloba*, known as *ginkgolides *[175]. In vivo, PAF is rapidly broken down by a specific acetylhydrolase, PAF-AH. Although not an eicosanoid, most PAF derives from the same enzyme system. Inhibitors of phospholipase A2, the rate-limiting step in the main route of PAF production, is under investigation as a drug target [176]. In EAE, elevated PAF in the spinal cord, which was not the result of reduced PAF-AH activity, appeared to be caused by cytosolic PLA2 activity [177]. For broader review of PAF as drug target, including for application to MS, see [178].

Evidence for PAF transport on platelet-derived microparticles (PMP) was reported [179], and more recently, the enzyme which degrades it, PAF-AH, was also identified on PMP [180]. PAF also circulates bound to plasma lipoproteins [181], is stabilized by albumin, and can be produced by many cells. Platelet activation induced by co-incubation with neutrophils stimulated by fMLP (fMLP stands for formyl-Met-Leu-Phe) was almost completely prevented by PAFR blockade; and the PAF released in the interaction was greater than the sum produced by platelets or neutrophils alone [182]. The explanation was that platelets secreted an inactive form of PAF (de-acetylated) which was re-acetylated by the neutrophils and then thrown back to strongly activate the platelets. This is consistent with our general hypothesis of the role of platelets in diseases such as MS: *that platelets are active partners with leukocytes *in their entry to the CNS. More specifically, the PAF secreted by the cooperation of platelets and leukocytes would facilitate opening the BBB in the microenvironment, since one of the most prominent actions of PAF is disruption of endothelial junctions [183-186].

Among the unexpected "pleiotropic" benefits of the statin drugs appears to be protection against neuronal damage caused by PAF [187]. It may be relevant that statin drugs have also been shown to inhibit release of endothelial microparticles [188], and this may apply also to platelet microparticles insofar as platelets have the same pathway of microparticle production.

A study of Japanese MS patients found that the PAF degrading enzyme, PAF-AH, was significantly lower in the patients [189] but this was not reflected in the genotype. On the other hand, the same group studied PAF receptor (PAFR) gene polymorphisms in MS and found significant differences compared to controls, concluding that this gene is a susceptibility factor for MS [190]. A gene microarray analysis of MS lesions revealed that transcripts for PAFR were among those elevated in chronic/silent plaques, as were the platelet-specific glycoproteins IIb and IIIa [191]. A few years later, Kihara et al reported that in an EAE model of MS, levels of mRNA for the PAF receptor (PAFR) in murine spinal fluid correlated with disease activity, and that knock-out of the PAFR gene resulted in lower incidence and abrogated severity of symptoms [192]. In earlier work, Callea et al reported 6-fold elevated PAF in the plasma of human RRMS patients and 15-fold elevation above controls in CSF [193]. Levels correlated with radiographic findings. The PAF subtype differed between plasma and CSF, indicating "different cellular origins" in the two compartments [193].

PAF can cause thrombocytopenia at levels as low as 3 ng/kg [194]. Mild thrombocytopenia is sometimes reported for MS patients (see later) and is possibly a signature of PAF activity. A role for PAF in stroke and brain injury has long been suspected [195]. Blockade of PAFR substantially reduced leukocyte adhesion to endothelia of hamster cheek pouch vessels following ischemia and reperfusion [196].Osborn et al explored the protective action of plasma gelsolin on lipid mediators and found that it modestly but significantly (p < 0.0001) inhibited LPA-induced platelet activation, but markedly inhibited PAF-induced platelet activation (>75% inhibition) at physiological gelsolin concentrations [197]. Thus, gelsolin may be an important natural modulator of PAF activity; plasma gelsolin is often reduced in association with disease [197].

### Others

This review provides only a sampler of agents of interest in each category. Several other classes of lipid mediators relevant to platelets in neuroinflammation are covered in the reviews cited earlier, e.g. the several phosphatidyl inositol phosphates (PIP's), sphingosines, and the recent discovery of the importance of palmitoylation. Of closely related interest are the many phospholipases whose activities govern most lipid mediators.

## Inflammatory agents of the coagulation system

In the last decade it has come to attention that several of the coagulation proteins are inflammatory or anti-inflammatory, and these are relevant to neurodegenerative diseases because they are implicated in MS (see later). They relate to platelets since the two systems go hand-in-hand: Activated coagulation stimulates platelets (e.g., thrombin) and activated platelets amplify coagulation.

### Fibrinogen

The emergence of fibrinogen as a potent neuroinflammatory mediator has been a surprise [198,199]. It has been identified in lesions of MS patients, as referenced in following sections.

### The protein C system

The protein C system acts to curtail thrombin generation, mainly by inactivation of activated FV, but is rather complicated owing to cofactors such as protein S, protein C inhibitor, thrombomodulin, the endothelial protein C receptor (EPCR), and other complexities [200]. Protein C itself is a vitamin K-dependent PL-binding protein, meaning that it can exert its function on activated platelets, but also on the endothelium *via *EPCR. Perhaps the most spectacular demonstration of its anti-inflammatory potential was the discovery that activated protein C (aPC) is an effective therapy for sepsis, regarded as a severe inflammatory state [201,202]. This is remarkable in view of the decades of failed efforts to treat sepsis. The anti-inflammatory efficacy of aPC appears to be largely independent of its anti-coagulant action. The use of aPC in MS has been little investigated, but proteomic analysis of MS lesions revealed protein C inhibitor [203], leading those authors to demonstrate substantial benefits of aPC in the EAE model of MS. Genc had previously made a good case for such investigations, citing relevant literature [204]. Thrombomodulin was also effective in an animal model of inflammation induced by lipopolysaccharide (LPS) [205].

### Contact proteins

The kinin-kininogen system (KKS) is most familiar for instigating the contact (or intrinsic) coagulation cascade [206] and participating in platelet aggregation [207]. Its components are known inflammatory mediators [208]. Of direct relevance to MS, it was recently shown that a kinin receptor is pivotal to T leukocyte recruitment to the CNS [209]. The KKS system overlaps somewhat with the complement system. According to Colman, a specialist in the KKS system [210], it came to light through investigations of snake bites, leading to the discovery of the vasoactive nonapeptide, bradykinin, released by cleavage of high-molecular weight kininogen (HMWK, a.k.a. HK). Bradykinin is a main target of the widely prescribed ACE inhibitors, which are proving to have unexpected neuroprotective effects, i.e. independent of blood pressure [211]. With regard to the role of platelets in the above-cited study [209], it has been shown that platelet-leukocyte interaction can occur *via *HMWK bridging from platelet GP 1bα to the CD11b/CD18 complex (Mac-1) on leukocytes [212]. The more conventional mode of interaction is between P-selectin of platelets and P-selectin glycoprotein ligand-1 (PSGL-1, CD162) on leukocytes, which can be effectively blocked by antibodies to the sialyl Lewis^x ^antigen [43]. Interestingly, PSGL-1 was recently reported to be the means of entry of the neurotropic enterovirus 71, and probably other neuropathic viruses of the same family [213], but those authors do not offer insight on how it then gains access to the CNS. In view of the foregoing, a possible role for platelets warrants consideration.

### Thrombin

Lastly, thrombin itself has long been recognized for its inflammatory actions, recently extended by the finding that it is required to initiate CCR2-dependent leukocyte recruitment, and that it is "the principal determinant of the outcome after vascular injury" in several animal models (LPS-induced endotoxemia, antibody-mediated graft rejection, carotid artery ligation) [214].

## Platelets and complement

The complement (C) system is a humoral arm of the innate immune system and can attack self-cells when marked by autoantibodies or due to defects in proteins that protect against C. It is a complicated system of circulating proteins, analogous to the coagulation system in that its activation involves a series or cascade of proteolytic conversions of zymogens to their active forms, resulting in several ultimate products, notably, the C5b-9 membrane attack complex which kills by punching holes in the target cell. The C system has several links to the coagulation system [215,216]. For example, protein S, a cofactor of the protein C system, is carried in circulation largely bound to C4 binding protein (C4bp), a complement component. It is well known that C is central to the pathology of many autoimmune and inflammatory disorders.

Platelets possess C receptors CR2, CR3, CR4, C1q, C1-inhibitor, and factors D, and H. Others are listed in some sources but are disputed as they may be acquired from plasma. Platelets are capable of deploying the lethal C5b-9 complex [217]. In addition, like other blood cells, platelets contain a set of membrane proteins which specifically protect them against autologous C-mediated attack, i.e. against self-injury by C [218]. These are CD55 (decay accelerating factor, DAF), CD59 (membrane inhibitor of reactive lysis, MIRL), and homologous restriction factor (HRF) [219,220]. Morgan mentions others less well defined. Recently, another such protective protein, Crry, was identified in murine platelets and erythrocytes [221,222], later extended by those authors to work on leukocytes [223], but Crry appears to be absent in humans. Defects in these proteins can result in pathology, e.g. paroxysmal nocturnal hemoglobinuria (PNH). We investigated CD59 on platelets from PNH patients and found levels ≈10% of normal, but findings on altered sensitivity to C-mediated lysis were inconclusive [224].

A recent finding of significance in neurology is that platelet-bound complement fragment, C4d, is a highly specific biomarker for systemic lupus erythematosus (SLE) and neuropsychiatric lupus [225]. Those authors propose this as a biomarker of cerebrovascular inflammation generally. Moreover, it correlated closely with positive lupus anticoagulant (LA) (p < 0.0001), and less well with positive anti-cardiolipin (aCL) (p = 0.035) [225]. Work by the same authors demonstrated that platelet-bound C4d was associated with ischemic stroke [226]. We have drawn attention to evidence for C-mediated injury in the neuropsychiatric aspect of anti-phospholipid syndrome, which can resemble MS, as referenced [66]. According to Roach et al, C5a signaling in macrophages is synergistic with PAF and with LPA [227].

Despite the presence of the above mentioned protective proteins, platelets are very sensitive to C-mediated attack. We have witnessed the serum-dependent fragmentation of platelets into microparticles when opsonized with an anti-platelet IgM [228]. However, platelets are not entirely defenseless against C, as it has been shown that attack complexes are selectively shed from the platelet membrane on platelet microparticles (PMP), allowing recovery of the parent cell [229,230]. Butikofer et al has shown that the microparticles released from erythrocytes are selectively enriched in proteins that protect against autologous C, at the expense of these proteins in the parent cell, and in their discussion cite evidence that same is true of platelets [231]. This implies that microparticle shedding sensitizes the remnant cell to C-mediated injury.

## New methods are expanding the inventory of platelet constituents

The list of active agents in platelets has been expanding in recent years by application of new technologies: proteomics, lipidomics, and mRNA transcript analysis. Hundreds of proteins were identified -- and many others were not identified -- in the supernatant of activated platelets [232-234]. Some 578 proteins were identified just in platelet microparticles [235]. Several proteomic studies of the whole platelet membrane were recently reviewed [236], with a special section devoted to proteins of the lipid rafts. (Lipid rafts are regions of the membrane which resist detergent solubilization and play critical roles in platelet physiology [237,238]). Raft regions are selectively shed with microparticles, e.g. [239].

The platelet "transcriptome" is also growing. Although platelets lack nuclei, thousands of transcripts have been identified in platelets, including coding for enzymes, interleukin receptors, etc., many of which were previously unknown in platelets, e.g. PEAR-1 [240-242]. Some of the same leaders in the field have sought to extend proteomic studies to include platelet-specific genes [243]. A recent paper claims to show that nuclear factor NFkB is not only present in platelet but active [244], which seems impossible since NFkB is a nuclear transcription factor, as discussed in an editorial [245]. The still-nascent field of lipidomics, which applies the same methods as proteomics (mass spectrometry) but to lipids, promises great advances in sorting out the myriad of bioactive lipids. Potentially important new platelet proteins continue to come to light, such as the septins, reviewed in relation to neurodegenerative disorders [246], and the vanilloid receptor (TRPV1) for noxious stimuli such as capsaicin [247] but also for several catechol amine metabolites such as homovanillic acid [248]. We shall cite the significance of some of this work to MS presently.

## Serotonin: a platelet-neuron nexus?

In 1985, Stahl pointed out a number of parallels between platelets and neurons, with focus on the storage and secretion of serotonin (5-HT), and the similar sensitivities to many CNS-active drugs [249]. Reed et al devoted a section of their review of mechanisms of platelet secretion to a comparison with vesicle trafficking in neurons [250], echoing work by Lemons et al [251]. Steidl et al in a study of CD34+ hematopoietic progenitor cells identified numerous ion channels, neuromediators, and other proteins previously assumed to be restricted to the CNS [252]. The reason for abundant acetylcholinesterase activity of erythrocytes (but not platelets [253]) remains a mystery attracting much interest [254-257]. The above-mentioned finding of endocannabinoid receptors, semaphorin A, monoamine oxidase (MAO), etc., on platelets is more of the same.

Serotonin (5HT) does not itself cross the BBB but some of its metabolites or precursors do, resulting in a degree of correlation between peripheral and CNS levels of 5HT [258]. Platelets are the main source of circulating 5HT but arteries and veins can act as reservoirs [259]. Of the several receptors for 5HT, platelets possess 5HT(1), as well as a serotonin transporter (SERT). Among the more surprising recent discoveries is the role of 5HT in regulating bone density [260].

Of direct relevance to inflammation is the demonstration that 5HT enhanced monocyte stimulation of CD4+ T cells and cytokine production following LPS exposure [261]. That paper also reports increased plasma 5HT in Alzheimer's disease (AlzD), which correlated with disability index [261]. Ciz et al reported inhibition of the oxidative burst of phagocytes by 5HT, *via *action on the 5HT receptor and on myeloperoxidase activity [262].

Treatment of MS with selective serotonin reuptake inhibitors (SSRI's) was reportedly very promising [263]. Plasma levels of 5HT are governed mainly by the number of SERT's on the platelet, with more or less of them appearing at the membrane surface depending on external 5HT concentration [264]. Accordingly, the number of SERT's per platelet is sensitive to SSRI's. Platelet SERT density correlated closely with P-selectin expression, a marker of platelet activation and cell-cell interaction, consistent with the concept of 5HT as a weak agonist [265]. However, Galan et al concluded that, contrary to tradition, 5HT is not a weak agonist of platelets, but instead sensitizes them or potentiates their responsiveness [266].

Abdellah et al observed significant differences in the action of SSRI on platelets, depending on polymorphisms of the gene for SERT (SLC6A4) and its promoter (5-HTTLPR) [267]. In an EAE model, it was found that knock-out of SERT caused increased plasma 5HT and attenuated symptoms, with effects most pronounced in female animals [268]. Interestingly, a strong gender effect was seen also with the influence of cannabinoids (in habitual cannabis smokers with cognitive impairment) on 5HT uptake in plasma [269]. In MS, levels of 5HT in CSF appear to correlate well with progression of disease [270]. Norepinephrine, at plasma levels seen in stress disorders, desensitizes the 5HT(1) receptor by uncoupling it from SERT *via *G proteins, leading the authors to conclude that NE can modulate 5HT responses and the action of SSRI's [271].

It was recently shown that 5HT can become covalently bound to a number of proteins, a process termed *seritonylation*, including to small GTPase's, with roles at the vessel wall [272] and in platelet activation [273]. The latter authors report that low levels of plasma 5HT markedly prolong the bleeding time. In summary, these brief notes from a large literature suggest that 5HT from platelets, or acting on platelets, may be a significant factor in neuroinflammatory conditions, especially in a restricted microenvironment such as at the BBB.

## Platelets and Alzheimer's disease: untangling the tangles

Although the focus of this review has been on MS, many of the papers cited refer also to other neuroinflammatory disorders. Here we take the example of Alzheimer's disease (AlzD). In 1998, we reported chronic platelet activation in AlzD [274] but that work was largely ignored in the blinding light of the β-amyloid hypothesis. Several recent developments are now forcing a "radical rethink" of the disease. (i) Platelet activation in AlzD has been confirmed [275]. Levels of homocysteine, considered a marker of hypercoagulable state, also impact on AlzD [276]. (ii) Two recent large genetic studies uncovered, in addition to APOE, three other AlzD-related genes, notably clusterin and CR1, both signatures of complement involvement [277,278]. (iii) Mounting evidence indicates that AlzD is inflammatory in nature and etiology [279], and that it can be controlled by exercise [280] and possibly diet [281]. (iv) Consistent with an inflammatory vascular etiology is the presence of complement fragment C1q in the brain lesions of a mouse model [282] and of the coagulation protein, fibrinogen, in human AlzD plaque [283]. (v) The hypothesis that age-related cognitive decline generally is mainly a vascular condition is supported by finding that retinal microvascular abnormalities predict decline [284]. (vi) To the extent that β-amyloid is truly causative, it should be noted that platelets are the principal source of amyloid precursor protein (APP) [285,286]. However, the relation of plasma levels of β-amyloid forms to AlzD has been controversial, and ratios of some of the forms are associated as much with vascular dementia as with AlzD [287]. In this connection, it is relevant to note that platelets appear to be responsible for post-surgical cognitive impairment [288].

Finally, (vii) recent work linking all of the above into one coherent hypothesis was brought to our attention during peer review of this paper: Platelets are now strongly implicated in the overexpression of the enzyme which liberates the offending peptide, amyloid-β (Aβ), from APP, known as BACE (*β*-site *A*PP *C*leaving *E*nzyme), or generically, "β-secretase". BACE was first identified as the long-sought β-secretase in 1999 [289] but those authors did not assay platelets in their study of its tissue distribution. In 2004, a direct relation was found between early-stage AlzD and platelet activity of BACE, as well as ADAM10, leading those authors to propose this assay as a diagnostic aid [290]. (ADAM10, also on platelets, has similar activity [291]). This was confirmed in 2005 [292] and again in 2008 [293]. BACE inhibitors to protect against progression of AlzD are in active development, but Hu et al advise caution because of their finding that BACE inhibition also delays (but does not totally abolish) remyelination by blocking cleavage of neuroregulins [294]. Interestingly, it was recently shown that platelet membrane cholesterol content modulates the activity of β-secretase, possibly explaining reported relations between cholesterol, dietary lipids, and AlzD [295].

## Platelets in multiple sclerosis: smoking gun?

MS is an immune-mediated demyelinating disease of the CNS and can be regarded as a model neuroinflammatory condition. Like most other autoimmune disorders, the etiology and pathophysiology of MS remains uncertain but most agree that a combination of genetic and environmental factors are required to initiate an immune reaction against CNS antigens. For example, Gong et al [296] has proposed a 5HT deficiency at high latitudes as a predisposing factor in the epidemiology.

The possible involvement of platelets in MS was first studied by Putnam in 1935, who considered a role for venule thrombosis in CNS demyelination [297]. In the 1950's-60's, at least ten studies appeared on the relation of platelets to CNS demyelination, several of which reported augmented platelet adhesiveness in MS, which correlated with disease activity [298-301]. More recently, a number of observations of platelet abnormalities in MS patients have appeared [302-304], and others cited below. We became interested when colleague W. Sheremata encountered MS patients with severe immune thrombocytopenic purpura (ITP) [305], leading to our report of increased platelet microparticles and platelet activation marker CD62P (P-Selectin) in MS [306]. Thus, chronic platelet activation in MS may now be regarded as well established, including by the report of Cananzi et al cited earlier [54]. Epidemiological studies have found a prevalence of ITP-like thrombocytopenia in MS patients about 25-fold higher than in the general population [304,307]. As earlier mentioned, mild thrombocytopenia, which could often be overlooked as insignificant, could be a signature of PAF activity.

It may be objected that a modest degree of platelet activation in MS is simply a consequence of general inflammation. However, the finding of platelet-specific GP IIb/IIIa in lesions of MS patients [191] comes close to "Smoking-Gun" evidence of platelet involvement. Relatedly, several elements of coagulation have been detected in the lesions including fibrin, tissue factor (TF), and protein C, suggestive of a procoagulant state [191,203].

For these and other reasons, MS has been described as a vascular disease [308]. The adhesion molecule, PECAM-1, may be particularly important in this regard. It was reported in 1999 that levels of serum soluble PECAM-1 (sPECAM-1) are significantly elevated in patients with active, gadolinium-enhancing lesions [309]. In 2001, we reported that PECAM-1-positive endothelial microparticles (EMP) are elevated in MS patients only during relapses, and correlated well with gadolinium-enhancing lesions [310]. In 2005, another study documented increased sPECAM-1 during acute relapse and in remission compared with progression [311]. It is not clear if the microparticle-bound form is functionally distinct from the soluble form but we have discussed examples of "soluble" biomarkers which are in fact membrane particle-bound [52] (Part D). These and other findings suggest that PECAM-1, whether soluble or MP-bound, may be at least an indicator of BBB disruption in MS and a biomarker of disease activity, and probably a key participant. The PECAM-1 gene is located on chromosome 17 in the region 17q23 [312] and, given that the region 17q22 was proposed to be an MS-susceptibility factor, PECAM-1 seemed to be a good candidate gene. However, at least two studies failed to find convincing support for that hypothesis [313,314].

Our findings, together with evidence reviewed in prior sections, suggest a broader hypothesis, namely, that platelet interaction with leukocytes at the endothelium of the BBB is responsible for the release of PECAM-1 to the circulation, and associated infiltration of leukocytes. Platelets are capable of directly activating both lymphocytes and dendritic cells [103,315]. In addition, it is tempting to postulate involvement of platelet activating factor (PAF) in view of its potency at disrupting endothelial junctions, and likely signature of thrombocytopenia (see Section 9). Elevation of PAF in the CSF and plasma of RRMS patients was reported, and the authors concluded that PAF is likely responsible for the early disruption of the BBB in MS [193]. Moreover, PAF receptors are up-regulated in MS lesions [191].

Differences in these parameters between RRMS and progressive MS have been noted in several reports, hinting at distinctive pathophysiologic pathways. Humm et al observed such differences in responses to prednisone [316]; and PAF activity was higher in RRMS than in secondary progressive MS [193]; and stronger differences were observed in sP-Selectin and other markers in RRMS compared to secondary progressive [311].

As earlier mentioned (Section 9), PAF is inactivated by PAF-AH but no association of the inactivating mutation of the PAF-AH gene with RRMS or progressive MS was found [189]. Nevertheless, the PAF-AH activities in MS were significantly lower than in healthy controls [189]. Such a decrease of PAF-AH activity may in part be responsible for the reported increase of PAF in MS plasma and CSF, and therefore could contribute to the inflammation and vascular permeability changes seen in the CNS of MS. On the other hand, a small Japanese study did find a significant association between MS susceptibility and a PAFR polymorphism causing a modest but significant reduction of PAF-dependent signaling [190].

We also found that platelet-associated IgM (but not IgG) is increased in MS patients [317]. We feel that this is of potential importance to understanding the pathophysiology of MS. However, present knowledge of antiphospholipid antibodies is too fragmentary to offer much insight on the significance of this finding, as discussed [66].

In summary, there are numerous mechanisms by which platelets could substantially contribute to the pathophysiology of MS. We do not pretend to have any specific hypothesis, nor do we propose that some bizarre platelet abnormality actually causes MS (although that is not impossible). Rather, the purpose of this review is to call attention to the neglected platelet and its potential to modulate inflammatory processes.

## Summary and conclusions

This review indicates that platelets could be pivotally involved with neurodegenerative and autoimmune conditions. At present, the potential role of platelets in such disorders has been neglected, although well appreciated in the cardiovascular and cancer research fields. The majority of studies in tissue culture designed to elucidate the pathophysiology of neurodegenerative diseases such as MS have investigated interactions of leukocyte subsets and endothelial cells, but it is likely that these interactions could be significantly modulated in the presence of platelets from patients *vs*. healthy controls.

The question may arise, exactly what kind of hypothesis could link platelets to such a wide variety of neurological conditions? Our view is that *platelets are partners with leukocytes and other immunological effectors *(complement, TLR's, PPAR's, resolvins, PAF, CD40/CD40L, etc.), amplifying or otherwise modulating those effectors in ways distinctive for each condition, with actions likely to be most prominent at the BBB.

A number of topics and many references were cut from this review because of excessive length. They include the role of platelets in several viral infections, consideration of other possibly relevant receptors (e.g., vanilloids, vasopressin), new work dissecting prothrombotic from inflammatory pathways, and further details on the topics covered. However, it is hoped that enough has been said to inspire new respect for the humble little platelet.

If this review succeeds in raising awareness of the potential roles of platelets in neurodegenerative and neuroinflammatory conditions, the labor of assembling it will have been amply rewarded.

## Competing interests

The authors declare that they have no competing interests.

## Authors' contributions

LLH, WJ, YSA, AHM, RZ, ME, JSA, and AM performed extensive literature research, prepared the manuscript and provided expertise in interpretation of data obtained from several sources.

AHM and ME wrote the section on platelets and multiple sclerosis.

JSA and AM reviewed the manuscript extensively and provided constructive comments to improve the quality of the manuscript.

LLH, WJ, YSA, RZ, and AM provided clinical expertise in various fields of neuroinflammation and improved the quality of the original manuscript.

All authors worked as team members to generate this extensive review.

All authors read and approved the final manuscript.
